# A Review of Storage Temperature Recommendations for Apples and Pears

**DOI:** 10.3390/foods12030466

**Published:** 2023-01-19

**Authors:** Robert K. Prange, A. Harrison Wright

**Affiliations:** 1 Special Graduate Faculty, School of Environmental Sciences, University of Guelph, Guelph, ON N1G 2W1, Canada; 2 Kentville Research and Development Centre, Agriculture & Agri-Food Canada, Kentville, NS B4N 1J5, Canada

**Keywords:** apples, pears, storage temperature, refrigerated atmosphere, controlled atmosphere, dynamic controlled atmosphere, chilling-sensitive, energy savings, pre-harvest effects, storage humidity

## Abstract

An exploration of the range of expert opinions on the optimum storage temperature for apples and pears in RA (refrigerated air), CA (controlled atmosphere), and DCA (dynamic controlled atmosphere) is provided, based on the accumulated postharvest data from the last 20 years. Apple cultivars have been divided into two storage temperature groups (0 to 1 °C and >1 °C), based on chilling sensitivity. Increasingly, gradual cooling, rather than rapid cooling, is recommended for apple cultivars, especially for chilling-sensitive cultivars. European pear cultivars are held at storage temperatures close to or just below 0 °C since they are not chilling-sensitive, and most cultivars require a cold temperature to induce ethylene production and ripening, especially if picked early for long-term storage. Asian pears apparently have higher temperature requirements in CA, compared with European pears. The temperature recommendations for RA and CA storage differ in some apple and European pear cultivars. In such cases, the CA recommendation is, on average, approximately 0.9 °C higher for apple cultivars and approximately 0.5 °C higher for pear cultivars, compared with RA. Research evidence suggests that some apple and pear cultivars can be stored at higher temperatures in DCA than in CA, and if the ethylene inhibitor, 1-methylcyclopropene (1-MCP), is applied in CA and/or DCA, leading to possible energy savings and quality benefits. A cool growing season may increase postharvest disorders, depending on cultivar and region. The store or packinghouse manager may choose to mitigate potential postharvest problems by maintaining the storage temperature at or above the temperature listed here and/or using stepwise (gradual) cooling. The storage temperature can affect the humidity and vapour pressure deficit (driving force) in the storage room. Altering the vapour pressure deficit controls the water loss in stored fruit, which can affect various quality parameters and the occurrence of several storage disorders.

## 1. Introduction

The most recent estimate of the total world fruit production was 883 million tonnes (t) in 2019 (https://www.fao.org/3/cb4477en/cb4477en.pdf, accessed on 16 December 2022). The world’s apple (*Malus domestica* Borkh.) and European pear (*Pyrus communis* L.) production was estimated at approximately 104 million t (81 million t apple plus 23 million t pear) in 2021 (http://www.wapa-association.org/asp/article_2.asp?doc_id=643, accessed on 16 December 2022), which is approximately 11.8% of the total world fruit production and second only to bananas and plantains (18%). Asian pears (Nashi) (*Pyrus pyrifolia* (Burm.f.) Nak., *P. bretschneideri* Rehder, or *P. ussuriensis* Maxim.) production is historically small compared with apples and European pears and reliable production values are not available.

Storing at the lowest acceptable temperature is an essential factor in extending the storage life of apples and pears [1,2]. If stored above this temperature, fruit senescence and associated fungal decay will shorten the storage period, and if stored below that temperature, fruit may freeze, or the fruit can develop chilling-induced disorders [1]. There are some cultivars of apples and Asian pears that are prone to chilling disorders and a solution for these cultivars is to store them at a higher temperature and/or use low-temperature conditioning, which is a gradual lowering of the temperature over a period of several days or weeks [3,4,5,6,7,8].

It should be noted that ethylene production is stimulated by chilling in some apple and European pear cultivars [9,10,11,12,13,14,15,16,17,18,19]. Induction of ethylene production, which hastens ripening in pears, can occur at temperatures ≤ 10 °C [9,15,19,20,21], but in at least one cultivar, Passe Crassane, it must be ≤5 °C [22]. Some apple cultivars, e.g., Golden Delicious, Granny Smith, Lady Williams, and Fuji, are also chilling-sensitive and can be induced to ripen by exposure to 0–10 °C in air [9,15]. Cold-induced stimulation of ethylene formation is due to the increased activity of both 1-aminocyclopropane-1-carboxylic acid (ACC) synthase and ACC oxidase, which then leads to increased ethylene production, especially when the fruit temperature is increased after removal from cold storage [12,14,15]. The low-temperature requirement varies with the cultivar, with early maturing pear cultivars having a shorter requirement, 0–20 days, while later-maturing cultivars require longer durations, >40 days. The low-temperature requirement in pears can be reduced by applying exogenous ethylene and/or delaying harvest [16,17,18,19,23,24,25].

Controlled atmosphere (CA) storage is based on the alteration and maintenance of gas composition different from that of air (78 kPa N_2_, 21 kPa O_2_, and 0.03 kPa CO_2_) in the storage atmosphere of the commodity. Ripening at the lowest acceptable temperature is delayed by CA conditions in almost all apple and pear cultivars, compared with refrigerated air (RA) conditions [1,8,10,16,26]. The major benefit of CA is derived from lowering the O_2_ concentration as low as possible without inducing undesirable anaerobic metabolism. A low O_2_ concentration inhibits respiratory metabolism and, if sufficiently low, it also inhibits ethylene biosynthesis and perception and ripening [8,26].

Almost all the information on the recommended storage temperature for apples and pears is for fruit stored in CA, with a limited number of temperature recommendations for storage in RA. Unfortunately, there is no central repository for the recommendations and the last summary was by Kupferman at the 8th International Controlled Atmosphere Research Conference [27]. He presented a summary of the most recent (to that date) controlled atmosphere (CA) recommendations for apples and pears. It included CA requirements for 32 apple and 9 pear cultivars, from 9 different growing regions. His summary identified the optimum storage temperature, atmosphere, and storage duration for each of these cultivars. Prange [8] has updated Kupferman’s CA storage recommendations with more recent data, including information on temperature and CA recommendations for 116 apple and 38 pear cultivars. The intent of this review is to provide more current storage temperature recommendations along with the factors identified by Prange [8] that can affect the recommended storage temperatures.

## 2. Development of a Database

A database of postharvest information generated between 2000 and 2022 was compiled from published and unpublished sources. Including the information from Kupferman [27], this database now comprises 116 apple cultivars and 42 European and Asian pear cultivars from approximately 17 growing regions (Table 1 and Table 2). Each recommendation in the database included most of the following key details: cultivar(s), country/region, year, optimum RA storage temperature, optimum CA storage temperature, recommended O_2_ and/or CO_2_ atmospheres, and storage duration, plus any relevant notes.

Since 2003 when Kupferman’s summary was published, the amount of information on CA recommendations for apple and pear cultivars has significantly increased. Not only has the number of cultivars greatly increased (Table 1 and Table 2), but the number of recommendations for each cultivar has also grown. For example, there are more than 35 CA recommendations each for Gala and Golden Delicious.

The increase in the number of cultivars in the database is a reflection of the changes in the apple and pear industry. Not only are there new cultivars every year, but in some cultivars, there is evidence that some newly identified clones may have real differences that necessitate different harvest and storage recommendations. These clonal differences need more research. Perhaps researchers could identify the clone as well as the cultivar that they are using in future research.

Another trend in the industry is the appearance of new ‘club varieties’ that are protected for a number of years under plant breeders’ rights in many countries. Even though growers are being encouraged to plant these club varieties, many of them could not be included in this database due to limited, or non-existent, harvest and storage information. Part of the club variety trend is the use of a new trademarked name (and sometimes multiple names) as an alternative to the actual cultivar name. The approach employed here is to use a generally accepted cultivar name and list in parentheses the known alternative names, e.g., Cripps Pink (Pink Lady).

## 3. Effect of Cultivar, CA, and RA on Storage Temperature Recommendations

Several trends since Kupferman’s publication can be identified by examining the postharvest database information. First, the apple cultivars listed in Table 3 and Table 4 can be divided into two groups based on their chilling sensitivity, using 1 °C as the critical boundary temperature. This is believed to be the first instance of grouping apples in this way:

- Chilling-insensitive: apple cultivars that are not chilling-sensitive in CA and can be stored at 0 to 1 °C (Table 3).
- Chilling-sensitive: apple cultivars that are chilling-sensitive in CA and need to be stored > 1 °C (Table 4).

**Table 3:** Apple cultivars that are not chilling-sensitive in CA (can be stored between 0 and 1 °C), arranged from lowest to highest recommended storage temperature. If the number of recommendations < 4, the CA temperature recommendation should be considered provisional (from Prange [8], with permission).

Chilling-Insensitive Cultivars (Including Clones)	Mean Recommended CA Temperature (°C)	Number of Recommendations
Mutsu (Crispin)	0	5
Delicious	0.22	17
Granny Smith	0.47	14
Lady Williams	0.50	2
Spartan	0.58	6
Gala	0.61	27
Golden Delicious	0.62	22
Stayman	0.62	2
Fuji ^a^	0.63	12
Rome (Rome Beauty, Morgenduft)	0.65	5
Northern Spy	0.67	5
Braeburn ^a^	0.74	15
Delblush (Tentation)	0.75	2
Jonathan	0.75	4
Cripps Red (Sundowner, Joya)	0.75	4
Jonagold’	0.84	16
Šampion (Champion)	0.84	5
Ligol	0.92	3
Gloster	0.94	4
Ladina	1.00	2
Topaz	1.00	2

^a^ Gradual cooling may be required.

**Table 4:** Apple cultivars that are chilling-sensitive in CA (must be stored > 1 °C), arranged from lowest to highest recommended storage temperature. If the number of recommendations < 4, the CA temperature recommendation should be considered provisional (from Prange [8], with permission).

Chilling-Sensitive Cultivars (Including Clones)	Mean Recommended CA Temperature (°C)	Number of Recommendations
Caudle (Cameo)	1.13	2
Cortland	1.16	9
Ambrosia	1.20	5
Civni (Rubens)	1.22	3
Elstar ^a^	1.23	12
Winesap	1.25	1
Pinova (Corail) ^a^	1.25	4
Coop 38 (Goldrush)	1.38	2
Alwa	1.50	2
Scilate (Envy) ^a^	1.50	2
Cripps Pink (Pink Lady) ^a^	1.67	12
Idared	1.77	10
Scifresh (Jazz) ^a^	1.85	4
Belchard	2.00	2
Empire	2.12	10
Arlet	2.25	2
McIntosh ^a^	2.79	5
Nicoter (Kanzi) ^a^	2.88	5
Honeycrisp ^a^	3.00	6
Cox’s Orange Pippin	3.50	5
Belle de Boskoop	3.96	6
Lobo	4.12	2
Bramley’s Seedling	4.38	2

^a^ Gradual cooling may be required.

Over the last 20 years, a trend for gradual instead of abrupt (static) cooling recommendations has developed, and this trend is likely to increase in the future. Although most of the cultivars listed in Table 3 and Table 4 have only static temperature recommendations, some now also have one or more recommendations to cool gradually (stepwise) to the final CA storage temperature (noted in the tables). Most of these cultivars are in the chilling-sensitive group, i.e., those that should be stored above 1 °C.

Interestingly, two of the cultivars in the chilling-insensitive group (Table 3), ‘Fuji’ and ‘Braeburn’, also have some gradual cooling recommendations. The collected recommendations for ‘Fuji’ (Table 5) and for ‘Braeburn’ (Table 6) illustrate the range of CA temperature recommendations for some cultivars. For both cultivars, there are several recommendations for storage above 1 °C, suggesting chilling sensitivity of the fruit grown in those regions. There are also some recommendations for gradual cooling and/or delayed introduction of CA for these cultivars (Table 5 and Table 6).

Unlike the apple cultivars, very few of the pear cultivars have more than four temperature recommendations (Table 7). Two pear cultivars, Conference and Doyenné du Comice, do have at least one recommendation to gradually cool. Perhaps further research will determine whether these two cultivars are chilling-sensitive and could benefit from a higher storage temperature. Unless and until that happens, the mean storage temperature recommendations for European pear cultivars range from −1.0 to +0.25 °C. In contrast, the recommendations for the four Asian pear cultivars average from 0 to +0.50 °C, i.e., slightly higher than for the European pears.

## 4. Comparison of Storage Temperatures in RA, CA, and DCA and Effect of 1-MCP

### 4.1. Temperature in RA vs. CA

Some of the entries in the database have only an RA or only a CA temperature recommendation. Nevertheless, among the apple cultivars, there are 109 recommendations, for 54 cultivars, that contain both RA and CA storage temperature recommendations. Among these:

- 63% of the recommendations specify the same RA and CA temperature
- 35% of the temperature recommendations are higher in CA than in RA, i.e., higher by a mean of 0.93 °C (range of 0.2 to 3.5 °C)
- 1% of the recommendations are lower in CA, i.e., lower by a mean of −0.40 °C

The higher temperature in CA is in agreement with Kupferman [27], who suggested that the CA temperature for a few specific apple cultivars can range from 0.5 to 1.0 °C higher than the RA recommendation. Interestingly, Fidler et al. [28] also stated that “the temperature used for a given cultivar kept in RA is usually 0.5 to 1.0 °C lower than that used in CA.” Two explanations are offered for this difference. Fidler, Wilkinson, and Edney [28] say that it is because “apples are more susceptible to low-temperature breakdown in CA than in air,” so it is necessary to use a higher temperature if CA is used. However, another more plausible explanation was offered much earlier by Davis and Blair [29] in a study on McIntosh, a chilling-sensitive cultivar. They state that using CA for this cultivar in combination with a non-chilling temperature, e.g., 4.4 °C, not only increases the storage period, compared with RA, but actually reduces the hazards associated with storage at a colder temperature.

For European pear cultivars, there are 34 recommendations for 17 cultivars that included both RA and CA storage temperature recommendations:

- 62% of the recommendations specify the same RA and CA temperature
- 38% of the recommendations are higher in CA than RA, i.e., higher by a mean of 0.52 °C (range of 0.1 to 0.7 °C)

There have been no studies of Asian pears that include both RA and CA storage temperatures. Therefore, at this time, it is assumed that the recommended storage temperature in RA and CA should be the same.

### 4.2. Temperature in DCA vs. CA

To date, commercial recommendations for storage temperatures in a dynamic controlled atmosphere (DCA) are the same as for conventional CA. However, physiological studies show that lowering the O_2_ concentration below 1.0 kPa, as in DCA, reduces apple and pear metabolic rates compared with the rates in CA (where typical O_2_ > 1.0 kPa). This allows storage temperatures in DCA to be higher than in CA, possibly up to approximately 5 °C, without incurring any negative metabolic effects (see Prange [8] for oxygen recommendations).

In support of this, the research on increasing storage temperatures in DCA, compared with CA, reports a number of possible benefits (Table 8). These include energy and cost savings, fewer storage disorders (especially chilling disorders), reduction in storage rot, less weight loss, and improved flavour. More research may be required to define the benefits of higher storage temperatures for specific cultivars of interest, perhaps also with higher commercial DCA temperature recommendations.

### 4.3. Effect of 1-Methylcyclopropene (1-MCP) on Storage Temperature Recommendations

The growth regulator 1-methylcyclopropene (1-MCP) is a vapour under physiological conditions and acts by inhibiting the binding of the hormone ethylene to its binding site [37,38]. Exposure to 1-MCP can temporarily render plant material insensitive to ethylene when applied at the parts-per-billion level. It is applied as a postharvest treatment but is sometimes applied on trees pre-harvest. The 1-MCP can reduce several serious storage disorders that cause fruit loss, such as senescent breakdown and superficial scald, but the incidence of others, including carbon dioxide injury and flesh browning, can be increased by 1-MCP [38]. Since apple fruit in storage can respond well to 1-MCP treatment, it is recommended for use on most apple cultivars, whereas recommendations for its use in stored pears has been more limited to a few selected cultivars.

Mir and Beaudry [39] were the first to propose the use of 1-MCP to reduce the requirement for refrigeration. They stored Delicious apples in RA at temperatures between 0 and 20 °C. They concluded that storage of 1-MCP-treated apple fruit at elevated temperatures will be limited to relatively short durations (<50 days) without some means of controlling storage decay.

Mattheis [40] stated that, in Washington State, some storage operators have altered CA conditions based on the reduced rates of fruit ripening after 1-MCP treatment. Higher O_2_ and temperature set points have been used in some warehouses, but widespread adoption of these changes has not yet occurred. The use of higher O_2_ and higher temperatures lowers the risk of low O_2_ or low-temperature injuries. An additional benefit is a decrease in power costs. McCormick et al. [41,42] examined the effect of adding 1-MCP to Gala and Jonagold apples in commercial rooms under CA conditions in Germany. The setpoint temperature of the control rooms without 1-MCP was 1.5 °C and, in the 1-MCP rooms, it was 2.5 °C higher at 4.0 °C. Assessment of the Gala and Jonagold fruit removed from storage at various times and evaluated for quality after seven days at ambient temperature to simulate marketing showed that 1-MCP-treated fruit at the higher storage temperature had similar or better quality attributes, compared with the fruit not treated with 1-MCP. An energy audit after 6 and 8 months showed that a 35% (‘Gala’ experiment) and 26% reduction (Jonagold experiment) in energy was achieved by storage at the higher temperature. In the UK, a trial with Gala apples using a storage temperature of 3.5 °C in the 1-MCP-treated CA room, compared with 0.5 °C in the control CA room, yielded similar results as in Germany [43,44]. Harz-Pitre [45] reported on similar commercial trials with CA-stored Golden Delicious, Granny Smith, Gala, Delicious, and Cripps Pink in South Africa. The 1-MCP-treated fruit were stored at 1.5 °C warmer than fruit not treated with 1-MCP. The report claimed that the findings at the various packhouses turned out to be excellent, with an average of 16% kWh saving for each degree Celsius of temperature increase. Kittemann, McCormick, and Neuwald [31] stored Golden Delicious, Jonagold, and Pinova for seven months in either CA at 1 °C without 1-MCP or CA at 5 °C with 1-MCP. Storing at 4 °C higher with 1-MCP resulted in an energy saving of 70%. The effect of storing in CA at 5 °C with 1-MCP on fruit quality and decay varied with the cultivar, with no consistent negative effects.

Despite these commercially sponsored results, there are few recommendations of higher storage temperatures when using 1-MCP. Zanella [45] suggests that CA storage of Cripps Pink in Italy is possible at 4 °C with 1-MCP, compared with 2.5 °C without 1-MCP. In Belgium, there are recommendations for CA with and without 1-MCP for five apple and three pear cultivars [46]. For the five apple cultivars, if 1-MCP is used, the CA storage temperature recommendation does not change for Gala and Greenstar, increases 0.2 °C for Pinova, and increases 0.5 °C for Golden Delicious and Jonagold. For the three pear cultivars, if 1-MCP is used, the CA storage temperature recommendation does not change for Comice, increases 0.2 °C for Conference, and increases 1.0 °C for Alexander Lucas.

It may be possible to achieve energy saving by increasing the storage temperature in CA if 1-MCP is used but there needs to be more research on how much of an increase is acceptable for specific cultivars in each growing region. Köpcke [33] examined the effect of combining 1-MCP with DCA on German-grown Elstar, Jonagold, and Gloster apples stored at 2.0, 3.5, and 8.7 °C for 140 days plus a 10-day shelf-life. Their overall conclusion was that the combination of 1-MCP and DCA storage is more favourable than 1-MCP or DCA alone, especially at higher storage temperatures. de Oliveira Anese et al. [34] tested this assumption with Brazilian-grown Galaxy Gala apples stored at 1.5, 2.0, and 2.5 °C in CA and DCA, with and without 1-MCP. They concluded that Galaxy Gala apples can be stored at more elevated temperatures (2.0 and 2.5 °C) under DCA, with no extra benefit with the addition of 1-MCP application. The 1-MCP was only beneficial if CA was used, rather than DCA. Vanoli et al. [47] conducted a similar study with Abate Fetel pear using 2 storage temperatures (−0.5 and 1 °C), 3 atmospheres (RA, CA, and DCA), and 2 storage times (20 and 28 weeks), with the aim of finding a better strategy to ensure ethylene-induced softening without inducing storage disorders. Although fruit treated with 1-MCP reduced or stopped softening, especially at −0.5 °C, they concluded that 90–95% of 1-MCP-treated fruit stored in CA and DCA was saleable, regardless of storage temperature and storage. They recommended that further research is needed to develop an optimum strategy.

These results suggest that further research is needed on the benefit of combining 1-MCP with DCA to allow for higher storage temperatures and thereby achieve greater energy savings.

## 5. Effects of Growing Season Temperature on Storage Temperature

Examination of the variations in storage guidelines employed around the world for widely grown cultivars reveals that the recommended storage temperature in production regions with a relatively warm growing season is often lower than the temperature recommended in regions with cooler climates. This is consistent with the findings of Wright et al. [48]. It suggests that lower growing season temperatures, especially during the latter part of the season, are associated with increased susceptibility to low-temperature breakdown (LTB) of apples in storage, which can be avoided by increasing the storage temperature [48,49,50]. For example, in Cripps Pink apples, fruit can develop two types of flesh browning, both of which vary depending on the heat units during the growing season (Table 9 and Table 10 [51,52]). Diffuse flesh browning (DFB) is a chilling disorder which behaves like LTB, i.e., it occurs when the growing degree days (GDD, 10 °C base temperature) are below 1100–1200, and it can be controlled with a higher storage temperature, e.g., 3 °C (Table 11). Radial flesh browning (RFB) is a senescent disorder that may occur in regions or seasons that accumulate between 1100 and 1800 GDD (Table 9 and Table 10 [53]), although there is no clear predictable relationship between RFB and GDD [53]. This is not surprising since senescent breakdown can occur in all apple cultivars and is affected by a number of factors [14,18]. In contrast with DFB, RFB is controlled by a lowered storage temperature, either by storing directly at 1 °C, or by stepwise cooling to 1 °C (Table 11). However, fruit from a region or season that accumulates >1700–1800 GDD has little or no risk of developing either of these disorders during storage.

If no local expert advice is available and the growing season is cooler than ‘normal’ for a cultivar that is prone to cool season disorders, the store/packinghouse manager should consider mitigating potential postharvest problems by avoiding storage temperatures below those listed in Table 9, and/or by implementing stepwise cooling over 1–2 months. Climate change, which is slowly increasing the growing temperature over the course of many decades, may alter what is a ‘normal’ growing season. However, this change is gradual, relies on averaging over the course of many disparate years, and can be associated with other changes, such as increased frequency of drought and extreme temperature events. This makes it difficult to predict how climate change will affect storage temperature recommendations and this remains poorly studied.

## 6. Interaction between Storage Temperature and Humidity

The rate at which water is lost from a single apple or pear fruit is a function of three factors, i.e., the surface area of the fruit, the vapour permeance of the fruit surface, and the difference in water vapour pressure between the inside and the outside of the fruit (see [8] for more detail). The difference in water vapour pressure between the inside of the fruit and the room air is also known as the driving force.

Water leaves the fruit in the form of water vapour. The water vapour pressure inside the fruit is assumed to be 100% RH at the temperature of the fruit. If the room air is also at 100% relative humidity (RH), then there is no difference, and thus no net loss of water from the fruit. Therefore, in theory, as much water enters the fruit as leaves it. In practice, however, the room air cannot be maintained at 100% RH because of the need to cool the fruit.

**Figure 1:**
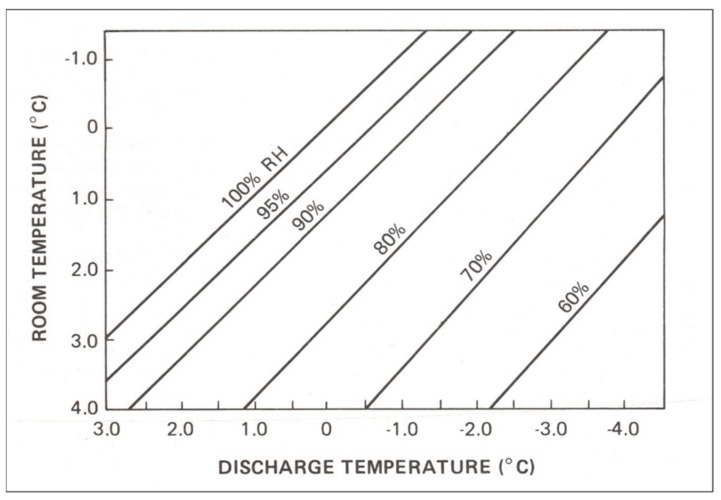
Relationship of the relative humidity (RH) of the room air to the temperature of the discharge air from the evaporation coils (from [8], with permission).

In all modern apple and pear storerooms, cooling is accomplished using the cooling coils of a mechanical refrigeration system to cool the room air and fans to distribute the cool air throughout the room. This type of cooling system increases the water vapour difference (driving force) because the cold temperature of the evaporation coils reduces the water-holding capacity of the room air. To achieve cooling, the temperature of the discharged air from the evaporation coils must be lower than the room air. This reduces its moisture-holding capacity and excess moisture condenses onto the evaporation coils. Thus, the relationship between the temperature of the room air and the temperature of the air discharged from evaporation coils dictates the maximum room air RH (Figure 1). Using the graph in Figure 1, one can estimate the maximum room air RH from two temperature measurements, i.e., room air and evaporation discharge temperatures, without measuring the actual RH in the room.

**Figure 2:**
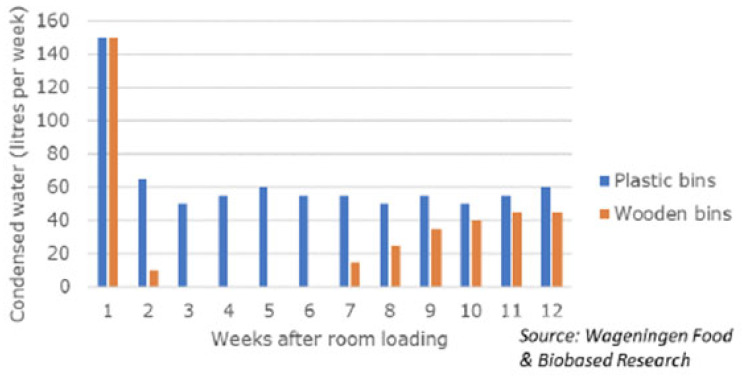
The amount of condensed water (litres per week) from a room with plastic bins and a room with wooden bins (150 tons of pears per room) (from [8,54], with permission).

During the room-filling stage and RA storage, the only reliable method to measure weight loss is by repeated weighing of fruit samples since the room humidity can be quite variable due to variable bin numbers, bin material (plastic vs. wood), and repeated room opening. For example, the use of dry wood bins, compared with plastic bins, results in a depression in storeroom humidity that can last for weeks (Figure 2).

**Figure 3:**
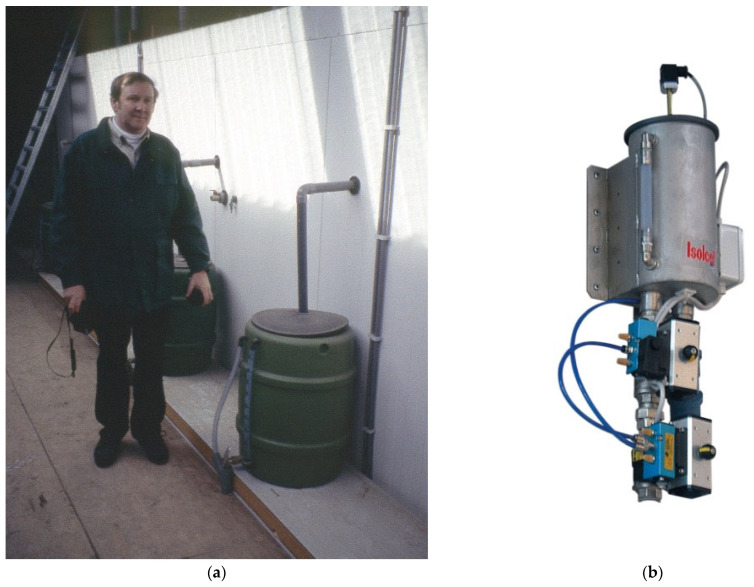
(**a**) Manual collection of water outside a CA room from the evaporation coils inside the room. There is a drain trap on the water pipe inside the CA room, to maintain airtight conditions. The volume collected in the green container is measured to determine moisture loss from the fruit, since 1 L collected equals 1 kg of weight loss from the fruit in the room. (**b**) Device for computerised electronic collection of water outside a CA room, from the evaporation coils inside the room (https://storage.isolcell.com/en/sentinel/). This system can redirect drainage water either back into the storeroom (onto the floor) or outside, according to the humidity requirements.

Note that, in a sealed CA room, one can measure the amount of condensation from the evaporation coils to determine if the room air RH is at its maximum. This can be measured manually (Figure 3a) or electronically (Figure 3b). If there is no condensate, then the room air is actually below the maximum possible RH (Figure 1). In such a situation, the fruit will be subject to greater weight loss. The RH can be increased by adding water to the room floor, or by misting, or by adding more fruit if the room is only partially filled. If condensate is present, the amount collected can be used to estimate weight loss in the storage room, assuming that 1 L of condensate water equals 1 kg of weight loss. This is expressed as litres/1000 kg/month, i.e., 1 L/1000 kg/month equals 0.10% weight loss per month.

Altering the water loss in stored fruit affects various quality parameters and the occurrence of several storage disorders [8,55]. Consequently, some specialists recommend an amount of weight loss during long-term CA storage which varies with the cultivar, e.g., 1.5–2.5% for Golden Delicious apple and Conference pear, 2.0–2.5% for Gala apple, and 2.5–3.0% for Elstar apple [55].

## 7. Conclusions

There is no single storage temperature that can be used for apples or pears. The choice of storage temperature is dependent on several factors. An exploration of the range of expert opinions on the optimum storage temperature for apples and pears in RA, CA, and DCA was provided in this study, based on the accumulated postharvest data from the last 20 years. Apple cultivars have been divided into two storage temperature groups (0 to 1 °C and >1 °C), based on chilling sensitivity. Increasingly, gradual cooling, rather than rapid cooling, is recommended, especially for chilling-sensitive cultivars. European pears are not chilling-sensitive and most of the cultivars can be stored between −1 and 0 °C. European pears also require a period of chilling, especially if harvested early, to stimulate ethylene production and ripening. There is limited information on the recommended storage temperature for Asian pear species. The current information suggests the storage temperature should be 0 to 0.5 °C in either RA or CA, with some cultivars needing gradual cooling to avoid chilling injury. The temperature recommendations for RA and CA storage differ in some cultivars. In such cases, the CA recommendation is, on average, approximately 0.9 °C higher for apple cultivars and approximately 0.5 °C higher for pear cultivars, compared with RA. Research evidence indicates that apples can be stored at higher temperatures in DCA than in CA, leading to possible energy savings and quality benefits. Applying 1-MCP to some cultivars of apples and pears may also provide similar benefits. A cool growing season may increase the chilling sensitivity and increase certain disorders, depending on the cultivar and region. The store or packinghouse manager may choose to mitigate potential postharvest problems by maintaining the storage temperature at or above the temperature listed here and/or using stepwise (gradual) cooling. The storage temperature can affect the humidity and vapour pressure deficit (driving force) in the storage room. Altering the vapour pressure deficit controls the water loss in stored fruit, which can affect various quality parameters and the occurrence of several storage disorders. 

## Figures and Tables

**Table 1:** Apple cultivars included in Kupferman’s work [27], and additional apple cultivars assembled from published and unpublished information available between 2000 and 2022 (from Prange [8], with permission).

32 Apple Cultivars with CA Recommendations, as Reported by Kupferman [27]	84 Additional Apple Cultivars with CA Recommendations
‘Boskoop’, ‘Braeburn’, ’Cortland’, ‘Cox’s Orange Pippin’, ‘Delicious’, ‘Elstar’, ‘Empire’, ‘Fuji’, ‘Gala’, ‘Gloster’, ‘Golden Delicious’, ‘Granny Smith’, ‘Gravenstein’, ‘Idared’, ‘Jonagold’, ‘Jonathan’, ‘Lobo’, ‘Macfree’, ‘McIntosh’, ‘Moira’, ‘Mutsu’, ‘Northern Spy’, ‘Nova Easygro’, ‘Novamac’, ‘Novaspy’, ‘Prima’, ‘Priscilla’, ‘Rome’, ‘Sciros’ (‘Pacific Rose’), ‘Spartan’, ‘Splendour’, ‘Stayman’	‘Alwa’, ‘Ambrosia’, ‘Antàres’, ‘Antonovka ohuknovenaya’, ‘Aport’, ‘Aprelskoe’, ‘Ariane’, ‘Arlet’, ‘Belchard’ (‘Chantecler’), ‘Belgica’, ‘Berkutovskoe’, ‘Bogatir’, ‘Bonita’, ‘Bonza’, ‘Bramley’s Seedling’, ‘Caudle’ (‘Cameo’), ‘Chopin’, ‘Choupette’, ‘CIV G198′ (‘Modi’), ‘Civni’ (‘Rubens’), ‘Coobishevskoe’, ‘Coolikovskoe’, ‘Coop 38′ (‘Goldrush’), ‘Corichnoe Novoe’, ‘Cosmic Crisp’, ‘Cripps Pink’ (‘Pink Lady’), ‘Cripps Red’ (‘Sundowner’, ‘Joya’), ‘Delblush’ (‘Tentation’), ‘Discovery’, ‘Egremont Russet’, ‘Firmgold’, ‘Glockenapfel’, ‘Golden Orange’, ‘Golden Russet’, ‘Honeycrisp’, ‘Imrus’, ‘Jonamac’, ‘Karmijn de Sonnaville’ (‘Carmine’), ‘Ladina’, ‘Lady Williams’, ‘La Flamboyante’ (‘Mairac’), Ligol’, ‘Macoun’, ‘Maigold’, ‘Martovskoe’, ‘Meridian’, ‘Milwa’ (‘Diwa’), ‘Nicogreen’ (‘Greenstar’), ‘Nicoter’’ (‘Kanzi’), ‘Noris’, ‘Orlik’, ‘Orlovskoe polosatoe’, ‘Pamyat Michurina’, ‘Pamyat Voinu’, ‘Pazazz’, ‘Pepin Shafrany’, ‘Pinova’ (‘Corail’), ‘Red Pippin’, ‘Reinette grise du Canada’, ‘Renet Chernenco’, ‘Renet Coorsky Zolotai’, ‘Rososhanskoe polosatne’, ‘Rubinette’, ‘Salish’, ‘Šampion’ (‘Champion’), ‘Scifresh’ (‘Jazz’), ‘Scilate’ (‘Envy’), ‘Severni Sinap’, ‘Sinap Beloruski’, ‘Sinap Orlovski’, ‘Skoroplodnoe’, ‘Spigold’, ‘Summerred’, ‘Tambovskoe’, ‘Topaz’, ‘Veteran’, ‘Vishnevonee’, ‘Welsey’, ‘Winesap’, ‘Worcester Pearmain’, ‘Yellow Newtown’, ‘York’, ‘Zhigulevskoe’, ‘Zimnee polosatoe’

**Table 2:** Pear cultivars included in Kupferman’s work [27], and additional pear cultivars assembled from published and unpublished information available between 2000 and 2022 (from Prange [8], with permission).

9 Pear Cultivars with CA Recommendations, as Reported by Kupferman [27]	29 Additional Pear Cultivars with CA Recommendations
‘Beurré Bosc’ (‘Kaiser Alexander’), ‘Beurré d’Anjou’, ‘Conference’, ‘Doyenné du Comice’ (‘Vereinsdechant’, ‘Sweet Sensation’, ‘Decana del Comizio’), ‘Forelle’, ‘Josephine’ (‘Joséphine de Malines’), ‘Packham’s Triumph’, ‘Rosemarie’, ‘Williams Bon Chretien’ (‘Bartlett’)	**25 European Pear cultivars** ‘Abate Fetel’, ‘Alexander Lucas’, ‘Alexandrine Douillard’, ‘Amfora’, ‘Angélys’, ‘Beurré Hardy’, ‘Cold Snap’ (‘Harovin Sundown’), ‘Concorde’, ‘Corella’, ‘Delbuena’, ‘Delmoip’, ‘Dr. Jules Guyot’ (‘Limonera’), ‘Erica’, ‘Flamingo’, ‘Forelle’, ‘Fred’ (‘CH 201′), ‘General Leclerc’, ‘Gute Louise’ (‘Louise Bonne d’Avranches’), ‘Harrow Sweet’, ‘Nojabrska’ (‘Novembra’), ‘Passe Crassane’, ‘Rocha’, ‘Selena’ (‘Elliott’), ‘Spadona’ (‘Blanquilla’), ‘Winter Nelis’ (‘Bonne de Maline’)
	**4 Asian Pear Cultivars** ‘Chojuro’, ‘Hosui’, ‘Nijisseiki’, ‘Ya Li’

**Table 5:** CA temperature recommendations for ‘Fuji’ apple (from Prange [8], with permission).

CA Temperature (°C)	Country	Year
−0.5	South Africa	2018
0	Poland	2016
	Chile	2020
0.5	Australia	2000
0–1.0	Canada	2012
	USA (California)	2000
0.5–1.0	Argentina	2012
	France	2010, 2014
1.0	USA (Washington)	2003
1.1 + gradual cooling	USA (Pennsylvania)	2008
2.0–2.5 + gradual cooling + delayed CA	Italy	2018

**Table 6:** CA temperature recommendations for ‘Braeburn’ apple (from Prange [8], with permission).

CA Temperature (°C)	Country	Year
−0.5	South Africa	2003, 2008
0.50	New Zealand	2003
0.50–1.0	Argentina	2012
	France	2010, 2014
	Switzerland	2018
1.0	Australia	2000
	Belgium	2017
	Poland	2016
	USA (Washington)	2003
1.1 + gradual cooling	USA (Pennsylvania)	2008
1.0–1.5 + delayed CA	Italy	2018
1.5–2.0 + delayed CA	UK	2017

**Table 7:** Temperature recommendations for European and Asian pear cultivars in CA, arranged from lowest to highest. If the number of recommendations is <4, the CA temperature recommendation should be considered provisional (from Prange [8], with permission).

Cultivar (Including Clones)	Mean Recommended CA Temperature (°C)	Number of Recommendations
European pears		
‘Angélys’	−1.00	1
‘Beurré Hardy’	−1.00	1
‘Dr. Jules Guyot’ (‘French Bartlett’, ‘Limonera’)	−1.00	1
‘Selena’ (‘Elliott’)	−1.00	1
‘Spadona’ (‘Blanquilla’)	−1.00	1
‘Abaté Fetel’	−0.62	4
‘Amfora’	−0.50	1
‘Delbuena’	−0.50	1
‘Delmoip’	−0.50	1
‘Erica’	−0.50	1
‘Forelle’	−0.50	2
‘Flamingo’	−0.50	1
‘Josephine’(‘Joséphine de Malines’)	−0.50	3
‘Nojabrska’ (‘Novembra’)	−0.50	1
‘Passe Crassane’	−0.50	1
‘Rocha’	−0.50	1
‘Rosemarie’	−0.50	2
‘Winter Nelis’ (‘Bonne de Maline’)	−0.50	1
‘Conference’	−0.49	12
‘Beurré Bosc’ (‘Kaiser Alexander’)	−0.47	8
‘Doyenné du Comice’ (‘Vereinsdechant’, ‘Sweet Sensation’, ‘Decana del Comizio’)	−0.44	10
‘Packham’s Triumph’	−0.43	10
‘Beurré d’Anjou’	−0.38	2
‘Williams Bon Chretien’ (‘Bartlett’)	−0.36	12
‘Alexander Lucas’	−0.25	2
‘Cold Snap ‘ (‘Harovin Sundown’)	0	1
‘General Leclerc’	0	1
‘Concorde’	0.12	2
‘Gute Louise’ (‘Louise Bonne d’Avranches’)	0.12	2
‘Fred’ (‘CH 201′)	0.25	1
Asian pears		
‘Ya Li’	0	1
‘Chojuro’	0.50	1
‘Hosui’	0.50	1
‘Nijisseki’	0.50	2

**Table 8:** Benefits of elevated storage temperature for apples stored in DCA (from Prange [8], with permission).

Cultivar(s)	Condition	Benefit	Reference
‘Royal Gala’, ‘Cripps Pink’ (‘Pink Lady’)	DCA-CF at 5 °C vs. CA at 3 °C	35% energy saving during cooling and 15% during storage.	[30]
‘Cripps Pink’ (‘Pink Lady’)	DCA-CF at 3 °C vs. CA at 3 °C	Reduced flesh browning.	[30]
‘Golden Delicious’, ‘Jonagold’, ‘Pinova’	DCA-CF at 3–4 °C vs. ULO at 1 °C	15–50% energy saving, less weight loss, less storage rot (Pinova), improved taste, no quality loss.	[31,32]
‘Elstar’, ‘Jonagold’, ‘Gloster’	DCA-CF (with or without 1-MCP) at 3.5–10 °C vs. ULO at 2 °C	Combination of DCA-CF +1-MCP is more favourable than either alone, especially at higher storage temperatures. Benefits include better firmness, and control of watercore, internal browning, and skin spots.	[33]
Galaxy Gala	DCA-RQ 1.3 and DCA-RQ 1.5 at higher temperatures (2.0 or 2.5 °C), compared with 1.5 °C	Galaxy Gala can be stored at higher temperatures (2.0 or 2.5 °C), because of lower mealiness, ethylene production, ACC oxidase, and higher flesh firmness, than at 1.5 °C.	[34]
Nicoter (Kanzi)	DCA-CF or DCA-RQ 1.5 at 3 vs. 1 °C	Both DCA methods at 3 °C produced better quality and fewer disorders, compared with 1 °C.	[35]
Royal Gala and Galaxy Gala	for both clones: 1.2 kPa O_2_ and 2 kPa CO_2_ vs. 0.8 k Pa O_2_ and 1.6 kPa CO_2_ for Galaxy only: 0.4 kPa O_2_ and 1.2 kPa CO_2_ at 1.0 vs. 1.5 °C	Storage of both Gala clones at extremely low O_2_ at 1.5 °C provided better quality, compared with 1 °C	[36]

**Table 9:** Relationship between growing degree days (GDD) from full bloom to harvest and the incidence of diffuse and radial types of flesh browning (FB) in Cripps Pink apples (from [8,51], with permission).

Type of Flesh Browning	GDD	Incidence of Flesh Browning, %
**Diffuse**	888	95.4
	888	99.4
	904	75.7
	904	76.0
	930	8.1
**Radial**	1462	57.5
	1462	54.2
	1567	27.8
	1641	10.0
	1679	0

**Table 10:** The effect of growing degree days (GDD) on the two types of flesh browning in Cripps Pink apples. The chilling disorder or diffuse flesh browning develops in cool regions, whereas senescent disorder or radial flesh browning develops in warm regions. Neither of these develop in hot regions. Although Cripps Pink is also susceptible to CO_2_ injury, this is not dependent on GDD (from [8,52], with permission).

District	GDD_10 °C_ (2005 Data)		Type of Flesh Browning		
			Radial	Diffuse	CO_2_ Injury
			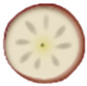	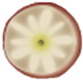	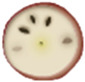
Tasmania, Australia	807	**Cool** 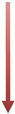 **Hot**		Y	Y
Nelson, New Zealand	1026			Y	Y
Hawkes Bay, New Zealand	1102			Y	Y
Yarra Valley, Australia	1162		Y	Y	Y
Manjimup, Australia	1405		Y		Y
Batlow, Australia	1556		Y		Y
Goulburn Valley, Australia	1688		Y		Y
California	1840				Y

**Table 11:** Overall recommendations for control of diffuse and radial flesh browning in Cripps Pink apples (from [8,51,52], with permission).

	Diffuse Flesh Browning	Radial Flesh Browning
**Classification**	Chilling injury	Senescent breakdown
**Climatic range**	<1100 GDD	>1100 GDD ^1^
**Maturity**	SPI ^2^ 3.5	SPI 3.5
**Storage temperature**	3.0 °C ^3^	1 °C ^4^ or stepwise cooling ^5^
**Storage atmosphere**	<1% CO_2_	<1% CO_2_
**Orchard management**	Ensure calcium levels are adequate	Best commercial practice ^6^

^1^ Since insufficient data in the climatic range of 1100–1400 growing degree days (GDD) > 10 °C are currently available, the type of flesh browning that develops in this range has not been determined. However, the general recommendations for radial flesh browning (RFB) may be suitable as a guide. ^2^ The starch pattern index (SPI) recommendation is based on the Centre Technique Interprofessionnel des Fruits et Légumes (CTIFL) 10-point scale. ^3^ Storage at 3 °C will prevent the development of diffuse flesh browning (DFB), however storage at 1 °C will reduce symptoms. Storage at 3 °C will reduce the period of storage before loss of quality occurs. ^4^ Storage at 1 °C successfully prevented RFB, however this was in a low-risk season. In a high-risk season, storage at a higher temperature may be required. ^5^ The stepwise cooling recommendation is: 2 weeks at 3 °C, followed by 2 weeks at 2 °C, then the remainder of the storage period at 1 °C. ^6^ Use best commercial practices for management of crop load and fruit nutrition.

## Data Availability

Not applicable.
